# The AI-driven blueprint: decoding intervertebral disc repair mechanisms for intelligent biomaterial design

**DOI:** 10.3389/fcell.2025.1751851

**Published:** 2025-12-11

**Authors:** Yuanzhen Shi, Yifan Wang

**Affiliations:** 1 Department of Orthopedics, The Second Hospital, Cheeloo College of Medicine, Shandong University, Jinan, Shandong, China; 2 Senior Department of Orthopedics, The Fourth Medical Center of Chinese PLA General Hospital, Beijing, China

**Keywords:** artificail intelligence, biomaterials, hydrogel, intervertbral disc, single-cell sequencing

## Introduction

The intervertebral disc (IVD), serving as a critical load-bearing structure connecting vertebral bodies, maintains spinal mechanical stability and function through the integrated health of its nucleus pulposus, annulus fibrosus, and cartilaginous endplates (Knezevic et al., 2021). However, intervertebral disc degeneration (IVDD) has emerged as a global health challenge, with its prevalence increasing markedly with age. As the leading cause of chronic low back pain, it imposes a substantial healthcare burden on society. Current clinical strategies for managing IVDD primarily focus on symptomatic relief—including physical therapy, pain management, and spinal fusion surgeries aimed at mechanical stabilization—none of which fundamentally reverse the degenerative process or achieve functional tissue regeneration (Copeland, 2007; Binch et al., 2021). With the expanding integration of artificial intelligence (AI) into medical science, particularly its growing capacity to uncover profound patterns within complex biomedical data, a new paradigm has emerged to address this challenge (Zhang et al., 2025). The application of AI in elucidating the mechanisms of IVDD and designing corresponding repair materials offers novel perspectives for regenerative rehabilitation of degenerative discs.

## AI for IVD repair mechanisms discovery

Artificial intelligence and machine learning are rapidly transforming intervertebral-disc research by integrating multi-omics data, single-cell profiles, and in-silico drug screening to decode the molecular logic of disc degeneration and repair. These algorithms outperform conventional statistics in ranking non-coding RNAs, programmed-cell-death regulators, or immune–matrix crosstalk genes, providing clinicians with quantitative biomarkers and druggable targets that can be validated *in vitro* and in rodent models (Liao et al., 2025).

The application of AI now extends to automating the diagnosis and grading of disc degeneration from lumbar MR images, where deep learning models demonstrate high precision in identifying pathological changes. Furthermore, single-cell RNA sequencing (scRNA-seq) has become a pivotal tool, revealing transcriptional shifts in disc resident and infiltrating cell populations following injury, and uncovering novel cellular targets for repair strategies. For instance, specific mesenchymal stem cell (MSC) populations were identified whose differentiation shifts with injury, offering potential for regenerative therapies (Lin et al., 2024; Stirnimann et al., 2025).

Li et al. introduced an RNA-seq-driven competing-endogenous-RNA strategy that couples differential lncRNAs, miRNAs and mRNAs in degenerated versus traumatic discs; they experimentally confirmed the XIST–miR-4775-PLA2G7 and XIST–miR-424-AMOT/TGFBR3 axes as pro-inflammatory circuits that disrupt extracellular-matrix homeostasis (Li et al., 2021). Zhang et al. presented a bioinformatics-plus-machine-learning pipeline that merges four GEO microarray sets, performs WGCNA–LASSO screening and constructs a protein–protein interaction network, identifying IL1R1 and TCF7L2 as central transcriptional hubs whose elevated expression distinguishes advanced Pfirrmann-grade discs with an AUC ≈ 0.7 (Zhang et al., 2024). Lv et al. delineated a comprehensive programmed-cell-death atlas of IVDD by integrating bulk and single-cell transcriptomes; machine-learning models selected PDCD6 and UBE2K as apoptosis-specific drivers, and *in vivo* administration of the repositioned drug Glibenclamide attenuated caspase-3 activity, preserved disc height and validated the predictive value of their ridge-regression score (Lv et al., 2025).

## AI for IVD repair biomaterials design

AI-guided single-cell multi-omics is advancing the rational design of biomaterials for intervertebral disc repair. By mapping cellular heterogeneity, cell death pathways, and fibrotic signatures at single-cell resolution, machine learning models enable the identification of therapeutic targets that are inaccessible via bulk profiling. These insights inform the engineering of mitochondria-targeting carriers, siRNA nanomotors, and immunomodulatory scaffolds—each mathematically optimized to maximize on-site efficacy while minimizing off-target effects. Convolutional neural networks cluster cell populations into distinct transcriptional states, whereas trajectory inference and random forest models predict lineage plasticity and gene significance. Meanwhile, reinforcement learning simulates the spatiotemporal behavior of biomaterials within a reconstructed disc microenvironment, iteratively refining their physical and biochemical properties to achieve an optimal therapeutic profile. This AI-driven approach transforms traditional “make-and-test” cycles into an in-silico “predict-design-validate” workflow, accelerating development, reducing animal use, and enabling patient-specific implants (Gao et al., 2022; Hu et al., 2023).

**FIGURE 1:**
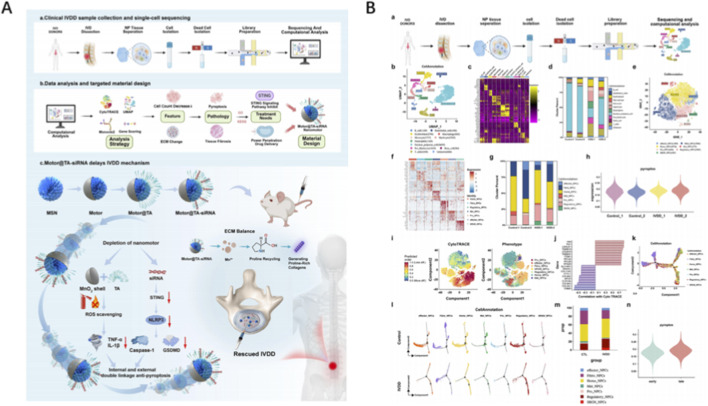
Application of AI in IVD repair biomaterials design **(A)** Schematic illustration of a single-cell-inspired self-enrichment pneumatic nanocarrier that inhibits pyroptosis to delay IVDD through intracellular and extracellular synergisms. **(B)** Single-cell analysis of nucleus pulposus tissues. Reproduced with permission from Copyright 2025 Wiley.

Tu et al. presented an atlas of human nucleus pulposus at single-cell resolution that resolves six NPC sub-states, fibro-progenitor trajectories and CD90^+^ progenitors with tri-lineage potency; bioinformatic deconvolution of immune infiltration revealed G-MDSC-mediated immunosuppression, inspiring a progenitor-enriched hydrogel that arrests fibrosis in rat IVDD (Tu et al., 2022). Zhou et al. introduced Motor@TA-siRNA, an H2O2-propelled nanomotor whose trajectory, siRNA load and tannic-acid shield were optimized by single-cell evidence of STING-driven pyroptosis; the carrier self-enriches within fibrotic NP, silences STING and simultaneously scavenges ROS, doubling disc height retention relative to passive vectors (Zhou et al., 2025) (Figure 1). Yang et al. described a deep-learning-assisted mitochondrial therapy in which scRNA-seq pinpointed OXPHOS-deficient fibrochondrocytes as the prime driver of fibrosis; exogenous mitochondria engineered with a mitochondria-targeting macromolecule PSP were shown to restore respiration, block mtDNA leakage and disrupt the SPARC-STING axis, achieving near-native NP architecture in a rat puncture model (Yang et al., 2025).

## Conclusion

In summary, AI is revolutionizing the study and treatment of intervertebral disc degeneration by enabling a shift from descriptive observation to predictive, mechanism-based intervention. It excels in decoding complex molecular mechanisms underlying IVDD, identifying critical biomarkers and cellular pathways through integrated multi-omics and single-cell analyses. In biomaterial design, AI accelerates the development of intelligent implants—such as targeted nanocarriers and mitochondrial therapies—by optimizing material properties and predicting therapeutic behavior through *in silico* modeling, thereby streamlining the traditional development pipeline.

Despite its promise, AI integration into disc research faces key challenges. The “black-box” nature of complex models obscures prediction interpretability, potentially limiting clinical adoption. Moreover, algorithm performance depends inherently on the quality, volume and diversity of training data; biases or noise in datasets compromise generalizability. Finally, translating *in silico* predictions and optimized biomaterial designs into safe, effective clinical applications remains a major hurdle requiring rigorous validation.

In our view, the full integration of AI into future intervertebral disc repair strategies is not only highly valuable but also an indispensable component. Conventional approaches struggle to address the complex etiological factors and significant inter-individual variability of IVDD. In contrast, AI provides a robust, data-driven framework for developing personalized therapeutic regimens grounded in solid mechanistic foundations. We should strive to build multimodal AI systems that integrate biomechanical, imaging, and clinical data to simulate the processes of disc degeneration and repair within a holistic digital twin framework. On the basis of interdisciplinary collaboration, we will advance these technological breakthroughs from theoretical concepts to clinical practice.
